# 4-Dimethylaminoantipyrine as a Broad Electrochemical Indicator for Immunosensors Platform

**DOI:** 10.3390/s22103681

**Published:** 2022-05-12

**Authors:** Francielli C. C. Melo, Renata P. Alves, Anderson L. Valle, Fabiana de A. A. Santos, Ana Carolina S. Dias, Isabela M. B. Goulart, Eduardo G. A. Oliveira, Guedmiller S. Oliveira, Luciano P. Rodrigues, Luiz R. Goulart

**Affiliations:** 1 Laboratory of Nanobiotechnology, Institute of Biotechnology, Federal University of Uberlândia, Avenida Amazonas s/n Bl. 2E, sl. 248, Uberlândia 38402-022, Minas Gerais, Brazil; francielli.melo@anvisa.gov.br (F.C.C.M.); anderson.valle@ibama.gov.br (A.L.V.); fabiana.aasantos@ufu.br (F.d.A.A.S.); ana.dias10@prof.edu.ma.gov.br (A.C.S.D.); 2 Biological Science, Universidade Federal do Triângulo Mineiro, Avenida Antônio Baiano, 150, Cidade Nova, Iturama 38280-000, Minas Gerais, Brazil; renata.pereira@uftm.edu.br; 3 National Reference Center for Sanitary Dermatology and Leprosy, Clinics Hospital, School of Medicine, Federal University of Uberlândia, R. Capricórnio, 94-Jardim Brasília, Uberlândia 38401-404, Minas Gerais, Brazil; imbgoulart@ufu.br; 4 Institute of Chemistry, Federal University of Uberlândia, Avenida João Naves de Ávila, 2121, Bloco 5T, sala 204, Uberlândia 38400-902, Minas Gerais, Brazil; eduardo.amaral@ufu.br; 5 Institute of Engineering Science and Technology, Federal University of the Jequitinhonha and Mucuri Valleys, Av. Um, n. 4.050-Cidade Universitária, Janaúba 39447-790, Minas Gerais, Brazil; luciano.rodrigues@ufvjm.edu.br

**Keywords:** immunosensor, 4-dimethylaminoantipyrine, electrochemical indicator, electrolyte, peptide probe, diagnosis

## Abstract

Here, we describe 4-dimethylaminoantipyrine (4-DMAA)-mediated interfacing as a broad biochemical indicator to stabilize and promote the higher response of electrodes for immunological detection. We hypothesized that the improved biological interactions of 4-DMAA with electrodes and biological samples may be due to the interaction properties of the benzene and pyrazole chemical groups with graphite and proteins, respectively. In order to demonstrate that 4-DMAA could be used as a general indicator in electrochemical immunoassays, we used peptides as probes for the diagnosis of four neglected tropical infectious diseases *Tegumentary leishmaniasis*, *Visceral leishmaniasis*, *Strongyloidiasis*, and *Leprosy* on commercial graphite screen-printed electrodes. 4-DMAA oxidation was used to indicate specific biological recognition between the epitope-based peptide and serum immunoglobulin G (IgG) from infected patients. We demonstrated that 4-DMAA should be incorporated into the electrodes prior to serum application, which avoids interference with its sensitivity and specificity. In addition, 4-DMAA oxidizes at a low anodic potential, and the oxidation peak is useful for detecting proteins in biological fluids. In summary, we have successfully demonstrated the broad application of 4-DMAA as a general indicator for the specific diagnosis of four infectious diseases in electrochemical immunosensors. Such a strategy is quite advantageous for indirect detection of proteins that lack electrochemical activities or are spatially inaccessible on the electrode surface. This new indicator opens a new avenue for monitoring biological recognition, especially for immunosensors.

## 1. Introduction

Electrochemical sensors have been the subject of intense research in recent years due to their wide range of use in diagnostics as well as in monitoring infectious diseases. Electrochemical indicators are commonly used in genosensors to mediate or display the hybridization processes of nucleic acids. These compounds are mostly planar aromatics, so there are several ways in which the compounds can interact with DNA by electrostatic interaction, groove binding, and intercalation [1]. In this context, many studies have reported useful DNA indicators such as ethidium bromide, acridine orange, methylene blue, hematoxylin, Hoechst dye, and tetramethylbenzidine [2,3,4,5,6,7,8]. However, it is difficult to find electrochemical indicators able to designate a biological recognition between proteins. Therefore, such indicators are explored here.

**Figure 1:**
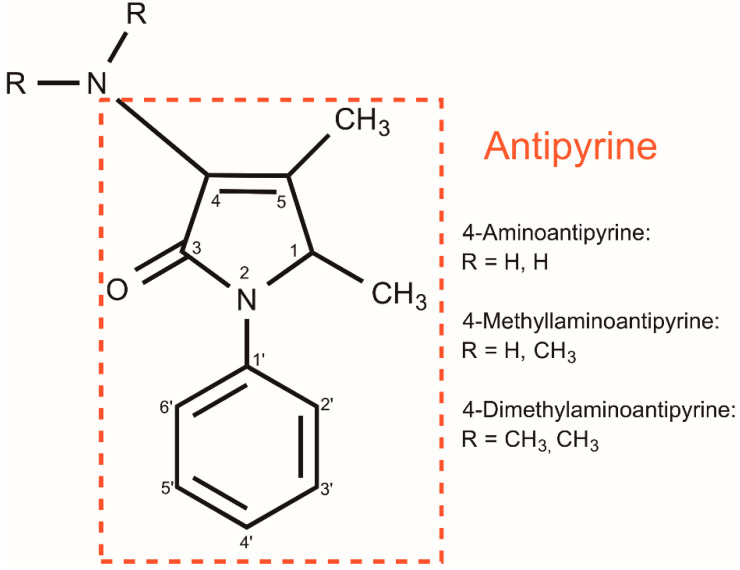
Structure of antipyrine and its derivatives.

The organic compound 4-dimethylaminoantipyrine or 4-dimethylamino-2,3-dimethyl-1-phenyl-δ3-pyrazolin-5-one (4-DMAA) is an active pharmaceutical ingredient that has long been used as an analgesic, antipyretic, and anti-inflammatory [9]. 4-DMAA can oxidize or reduce depending on the conditions of physiological systems, leading to the formation of radicals that appear to have high toxicity to the human body [9,10,11]. 4-DMAA, as 4-aminoantipyrine (4-AA), and 4-methylaminoantipyrine (4-MAA) are active metabolites with antipyretic action that has an affinity for biomolecules as dipyrone has [10,12]. It is expected that the toxic analog 4-DMAA may also exhibit a similar affinity for organic compounds due to its structure containing a pyrazole group (Figure 1). We hypothesize that the affinity of 4-DMAA for proteins along with its redox properties may be important traits to consider as a new indicator for electrochemical immunosensors. 4-DMAA was measured by HPLC with detection by amperometry, voltammetry, and NMR. GC–MS was also used to detect 4-DMAA in cocaine samples, which was used as an adulterant [13,14]. In this investigation, the oxidation of 4-DMAA was used as an electrochemical indicator to enable the recognition of epitope-based peptides and immunoglobulin G (IgG) by voltammetry. We believe that 4-DMAA is one of the first general electrochemical indicators for broad immunosensor applications that use proteins or peptides as recognition elements.

## 2. Materials and Methods

### 2.1. Chemical and Biological Reagents

All reagents used were of analytical grade without further purification. All experiments were performed at room temperature (25 ± 1 °C). Ultrapure (Type 1) water quality (Direct-Q^®^3 Water purification System, Merck Millipore) was used for the preparation of aqueous solutions. Phosphate-buffered solution (PB) 0.1 mol.L^−1^ was prepared at pH 7.4. Stock solutions of the probe (peptide), target (serum), and block solution (Bovine Serum Albumin (BSA)) 0.5% were prepared in PB and stored in a freezer until use. The probe and the serum activity were previously confirmed by ELISA before each test. The new electrochemical indicator, 4-dimethylaminoantipyrine (4-DMAA), was prepared in water (5 mmol.L^−1^) and stored at 4 °C in an amber flask (to prevent degradation by UV radiation) until its use. Due to noninterference in the three-dimensional structure of the probes, we chose to keep all solutions at physiological pH.

For specific biological recognition, we used four peptides related to neglected diseases with different etiological agents: protozoa, nematoda, and bacteria. These peptides were based on epitopes originated from phage display selections that mimic highly specific antigens of the target pathologies, as the probes *Visceral leishmaniasis* (VL)-LC1 (*Leishmania infantum chagasi*) and *Tegumentary leishmaniasis*-LT (*Leishmania amazonensis*), D3 for *Strongyloidiasis* (*Strongyloides stercoralis*) and M3R for leprosy (*Mycobacterium leprae*), and chosen based on previous diagnostic parameters obtained from serological tests, Enzyme-Linked Immunosorbent Assay (ELISA), which detected IgG patients serum samples. Negative and positive sera were confirmed by the gold standard assay. The ratio of the probe and target titers previously used in the ELISA immunoassay was calculated and maintained (Table 1).

### 2.2. Electrochemical Apparatus

Electrochemical voltammetric measurements (Differential Pulse Cycle) were performed using the PalmSens 3 potentiostat (PalmSens Compact Electrochemical Interfaces, Houten, The Netherlands). The differential pulse voltammetry with basic parameters used for the readings were: modulation amplitude (E pulse): 0.015 mV; pulse interval (t pulse): 0.06 s; sweep rate: 15 mV.s^−1^ (0.002 V step); current range: 1 nA to 10 mA; time balance: 2 s; potential: −0.2 V to 0.9 V. The electrochemical screen-printed electrodes (DropSens, Asturias, Spain) on the ceramic substrate: L33 × L10 × H 0.5 mm, consisted of the working electrode (4 mm diameter), counter electrode, and reference electrode, with the following shapes: type 1-carbon, carbon, silver (DropSens, Spain; ref. C110), and type 2-gold, gold, silver (ref. BT 220).

### 2.3. Conjugation of the Probe and Electrochemical Measurements

First, two 2 μL solutions of the probes were applied to the surface of the working electrodes and incubated for 30 min. The electrode was then washed using a p1000 pipette with 1 mL of phosphate-buffered saline and dried in manual air insufflation equipment. To block the binding of nonspecific biomolecules, 2 μL of 0.5% BSA for 20 min was used. Then, the electrode was again washed with 1 mL of phosphate buffer and dried in the same way as in the first step. After these two steps, the correct order of indicator (4-DMAA) addiction in the working electrode was tested: before the addition of serum and after the inclusion of the patients’ serum. Furthermore, 2 µL of 4-DMAA and 2 μL of serum were applied to the electrode surface for 20 min and 30 min, respectively, washed and dried in the same way as in the first step after each step, and its interaction with a probe was evaluated by differential pulse voltammetry (DPV). DPV measurements on the electrode connected to a potentiostat were obtained using 100 µL of phosphate buffer at pH 7.4 as the electrolyte to evaluate the electrochemical signal (by indirect detection). All electrochemical tests were performed in triplicate. All incubation periods were performed in the absence of light at room temperature.

### 2.4. Immunosensing Tests

We used sera from infected patients (VL-positive) to demonstrate the specific binding of the peptide-IgG complex (target probe). After immobilizing the biological probes on the surface of the working electrode, 2 μL of serum was applied to the carbon working surface for 20 min at room temperature. In the functionalization for the 4-DMAA test, when serum was added after or before this indicator, we used phosphate buffer (pH 7.0) as the electrolyte. The negative control (negative VL) was performed using newborn serum negative for the pathologies studied. The best results for the LC1 probe were repeated using different probes to confirm the biological recognition and the action of 4-DMAA as an electrochemical indicator.

### 2.5. Atomic Force Microscopy (AFM) Topographical Analysis

Atomic force microscopy (AFM) imaging was evaluated (SPM 9600, Shimadzu, Kyoto, Japan) to determine topographic changes before and after in the pre-and-post-immunoagglutination complex formation of the electrode surfaces. Roughness (Rq) analyses were performed on bare graphite electrodes (C110), C110 electrodes with 4-DMAA, C110 electrodes functionalized with the peptide LC1, followed by 4-DMAA incorporation, and then (LC1 + 4-DMAA) with IgG-positive sera and (LC1 + 4-DMAA) with IgG-negative sera.

## 3. Results and Discussion

### 3.1. Surface Interactions

**Figure 2:**
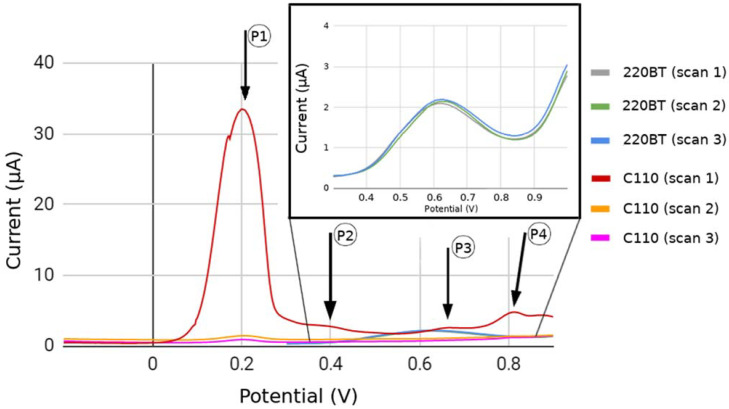
DPV for the first three scans of 4-DMAA (5 mmol.L^−1^) on the screen-printed graphite electrode C110 and screen-printed gold electrode BT220 in phosphate-buffered electrolyte salt solution (pH 7.4). P1, P2, P3 and P4 are the four oxidation peaks from 4-DMAA obtained using the graphite electrode.

Through the addition of hydroxyls to the aromatic ring, the 4-DMAA is able to form hydroxylated derivatives and oxidative demethylation, converting to 4-methylaminoantipyrine and 4-aminoantipyrine sequentially when in a buffered salt solution. The oxidation process of 4-DMAA depends on the pH of the solution, being accelerated by pHs closer to neutrality [9]. This is an important feature that gives 4-DMAA new possibilities for interaction with biomolecules. A study to verify the electrochemical behavior of metamizole (dipyrone) and other pyrazolones showed that 4-DMAA has up to four oxidation peaks. The peak refers to the loss of the first electron on the nitrogen atom that lies outside the pyrazole ring promoting the formation of a radical cation. The stabilization of the oxidation products in peak two occurs due to hyperconjugation of the iminium ion formed [13]. Another proposed oxidation mechanic of 4-DMAA involves a deprotonation step of methyl and carbene formation. It then loses two electrons to form a cation that is hydrolyzed to form the final product [15]. The first three scans of differential pulse voltammograms 4-DMAA embedded on the surfaces of carbon and gold electrodes, respectively, are shown in Figure 2. 4-DMAA was almost fully oxidized at the potential of 0.2 V (P1) in the first scan of the graphite electrode. Still in the same scan, 3 more less-intense peaks appeared for 0.4 V (P2), 0.65 V (P3), and 0.82 V (P4). A different behavior was observed on the gold surface, where we can observe a small gradual oxidation at a higher anodic potential of 0.63 V in three scans. 4-DMAA did not interact well with the gold surface, showing a more effective adsorption on the carbon surface, possibly due to van der Waals interactions between the graphite carbon and the 4-DMAA aromatic rings. For this reason, the other tests in this study were performed on a graphite electrode. Gowda et al. (2015) [16] evidenced the same behavior for 4-AAP on surface electrodes modified with multi-walled carbon nanotubes in cyclic voltammetry. 4-AAP completely and irreversibly oxidized at the potential of 0.512 V. In this case, the authors attributed the complete oxidation to the large surface area of the modified electrode.

### 3.2. Assembly of Bioelectrodes and Importance of the Immobilization Sequence of 4-DMAA on the Surface for Detection

**Figure 3:**
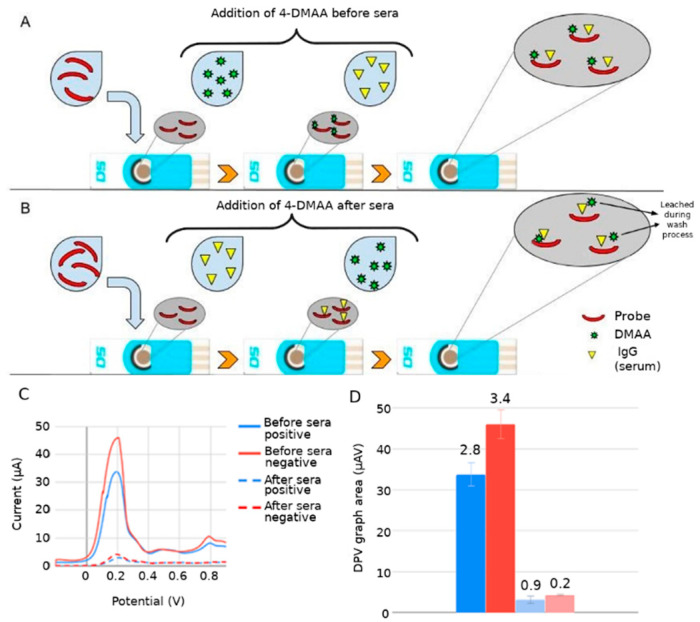
Differential pulse voltammetry (DPV) electrochemical results for the detection of Leishmaniasis using LC1 peptide and 4-DMAA at two different times of functionalization of the electrode surfaces. (**A**) Scheme of functionalization using 4-DMAA before serum; (**B**) scheme of functionalization using 4-DMAA after serum; (**C**) DPV peaks: 45.96 μA × 0.20 V; 33.73 μA × 0.19 V; 4.12 μA × 0.19 V; 2.92 μA × 0.21 V; and (**D**) DPV graph area (numbers at the top of histogram boxes are standard deviation values). Electrolyte: phosphate-buffered saline solution (pH 7.4).

Functionalization of the recognition surface begins after immobilization of the visceral leishmaniasis antigen (LC1) in the area corresponding to the working electrode (C110). Immediately afterward, age blocking of the free sites on this surface was performed with the addition of bovine serum albumin (BSA) to prevent nonspecific binding of the target. We incorporated 4-DMAA under two different biosensor assembly conditions: before serum addition and after serum inclusion. The best conditions for IgG-positive serum recognition were obtained when 4-DMAA was added prior to serum introduction, showing more significant differences between positive serum and controls (negative serum and peptide alone/other pathologies), and also larger anodic current peaks. Comparative indirect detection tests with 4-DMAA incorporated before or after serum showed significant differences in electrochemical results (Figure 3).

**Figure 4:**
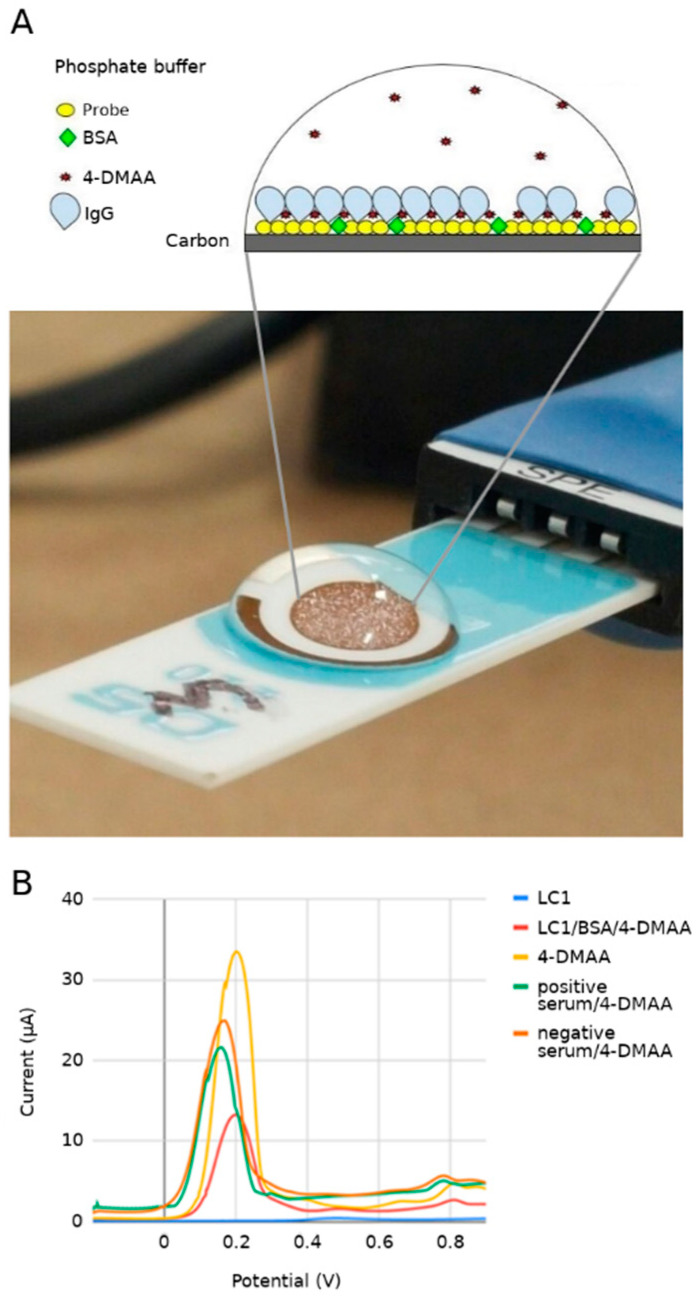
(**A**) Adsorption phases in sequential functionalization steps showing the DPV curves for each component added to the system. (**B**) Peak values for 4-DMAA: 33.50 μA × 0.20 V, negative serum/4-DMAA: 24.95 μA × 0.17 V, positive serum/4-DMAA: 21.60 μA × 0.16 V, LC1/BSA/4-DMAA: 13.25 μA × 0.19 V, LC1: 1.61 μA × 0.5 V. Electrolyte: phosphate-buffered saline (pH 7.4).

We observed that the amplitude of 4-DMAA oxidation peaks increased more than 10-fold when comparing the functionalization with the addition of 4-DMAA after and before sera (45 vs. 4.2 μA for negative serum, and 32 vs. 2.5 μA for positive serum) (Figure 4A,B). This difference is evidence of indicator predilection for the first biomolecules added to the carbon surface (peptide and BSA) and the weak interaction with IgGs and other serum components. In this case, the final washing of the electrode before voltammetry in phosphate buffer would remove much of the 4-DMAA deposited as the last functionalization step. This did not occur when added after the probe and blocker. Therefore, inserting the 4-DMAA before the addition of serum into the system was considered the best test condition for use in subsequent assays.

In Gowda et al. (2014) [17], it was demonstrated that the electrochemical signal in cyclic voltammetry and the fluorescence intensity of human albumin (HSA) decreased in accordance with increasing amino antipyrine (AA) concentration, with a greater perturbation of tryptophan, indicating a binding between 4-AA and HSA. Teng et al. (2011) [18] proposed that the interaction between BSA and AA occurs at negatively charged amino acid residues, such as Glu and Asp, through electrostatic and van der Waals forces. Teng et al. also proposed in 2010 that in addition to van der Waals forces, hydrogen bonds are also involved in the AAP–protein interaction, in this case, hemoglobin, which is a blood metalloprotein. Further supporting evidence was provided by molecular coupling and dynamics studies, which are of high affinity between pyrazole groups and like proteins [19]. The first peptide used, LC1, showed a negative electric charge (−2) at neutral pH, with an isoelectric point at 4.04. In its structure, two glutamates and one aspartate were shown, corroborating the dynamics of the amino antipyrine 4-DMAA.

The immunological detection of C-reactive protein using amino antipyrine as an electrochemical indicator showed that this indicator intercalates into aromatic amino acids by van der Waals forces in proteins [20]. The specific target recognition reaction by the probe likely altered the dynamics of electron passage at the electrode surface, generating a significant reduction in the stray current in the system, which did not occur in the voltammetric readings of the negative sera, which showed higher current peaks than the positive sera. Figure 4A demonstrates the theory of trapping the electrochemical indicator between the probe/blocker and the target, causing an insulating effect that reduces the oxidation of the electrochemical indicator. The lack of recognition between the biological and negative probe sera exposes the 4-DMAA adsorbed on the peptide and BSA, generating larger anodic peaks.

When we look at the graph of individual results for each adsorption phase, we can verify the electrochemical behavior after the addition of each biological or chemical component through VPD curves obtained from the experiments (Figure 4B). The LC1 probe (green line) in the absence of 4-DMAA shows a very low current reading near the baseline (phosphate buffer), defining the probe as a nonelectric donor element to the system, or even preventing the electric current [21]. Although it is negative at neutral pH (−2), the peptide in question has a significant number of hydrophobic amino acids that can further hinder the passage of electrons through the working electrode. 4-DMAA, on the other hand, shows a peak current, which is related to its oxidation, 30 times higher than the probe reading (orange line). When it is deposited on the probe and the blocker (red line), an oxidation peak appears between pure 4-DMAA and the peptide probe, indicating its adsorption on the carbon surface. When sera are added, the anodic current peaks decrease further, proving the interference of target probe recognition on the oxidation of our electrochemical indicator, as already discussed. Even in the oxidation curve of the negative serum control, there is a decrease in the amplitude of the peaks, indicating that there is still some nonspecific interaction on the surface of the system, despite the blocking and washing steps used.

The possible interaction of 4-DMAA with BSA should not disable its use as an indicator of biological recognition between proteins, as this occurs at the same intensity for both controls (positive and negative). Therefore, the 4-DMAA molecule exhibits redox properties and the ability to interact with both graphite and proteins being suitable to indirectly obtain a biological recognition signal between complementary proteins. For this study, when peptide-IgG recognition occurs, 4-DMAA is less exposed and lower oxidation current values are obtained. In contrast, when peptide-IgG recognition does not occur, 4-DMAA is more exposed for oxidation and higher current values are obtained. The difference between the two biological recognition states (on-off) can be indirectly indicated by the observed difference in the oxidation current of 4-DMAA.

### 3.3. Affinity of 4-DMAA by Different Peptide Probes

**Figure 5:**
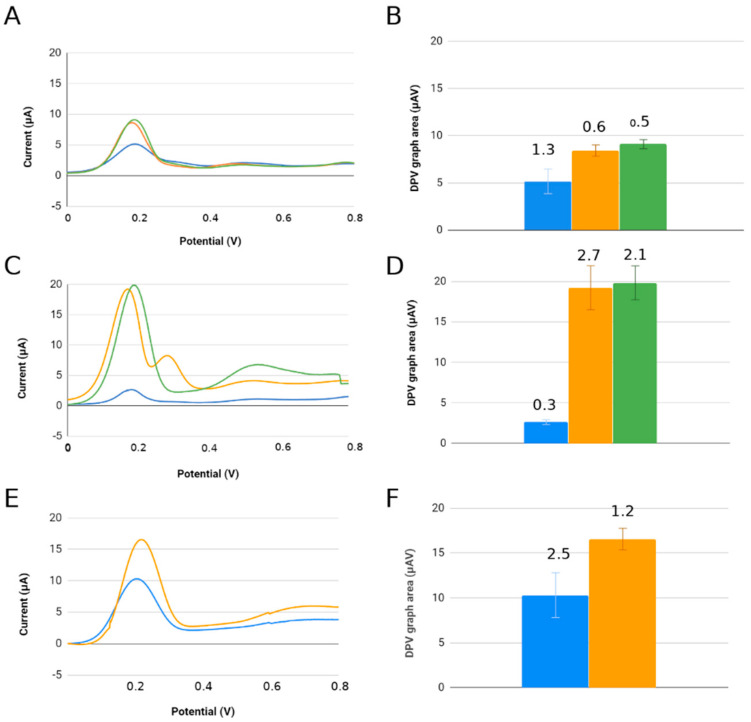
Results of differential pulse voltammetric readings using 4-DMAA before serum: (**A**,**B**) strongyloid D3 probe (peaks 9.09, 8.91, and 5.15 μA); (**C**,**D**) tegumentary leishmaniasis LT probe (peaks 19.84, 19.14, and 2.62 μA); (**E**,**F**) leprosy M3R probe (peaks 16.55 and 10.30 μA). Bars: blue = serum-positive, yellow = serum-negative, and green = controls (control was absent in F). Graphs showing current range −5 to 20 μA and potential 0 to 0.8 V (same scale for all probes). Electrolyte: phosphate-buffered saline (pH 7.4).

By using this test condition for the adsorption ordering steps on different peptide probes, we found that 4-DMAA is again able to distinguish between recognition of the target probe (D3-specific IgG strongyloid peptide) and nonrecognition of negative sera and other parasites by the probe. The same occurred with the LT recognition probe, according to Figure 5. Note that the concentration of the D3 and LT probes interferes with 4-DMAA anodic oxidation intensity peaks obtained in differential pulse voltammetry. For the lower-concentration probes, the total oxidation of this indicator occurred at lower currents, perhaps due to the lower amount of 4-DMAA adsorbed in the system, highlighting the need for higher probe concentrations for better electrochemical indicator fixation and consequently better detection of the IgGs under study. The LT and D3 probes have 24 amino acid residues and have an almost zero charge at neutral pH (LT = 0; D3 = −0.3), while the M3R probe is a 77-amino-acid-residue protein with a negative charge (−1.7 at neutral pH), which prevents a better comparison with the results of the other probes. Although its concentration was the lowest among the probes studied, its size was probably an important factor in immobilizing a good amount of the 4-DMAA indicator to obtain satisfactory anodic peaks in the identification of antibodies to M. leprae. Enache and [22], in a specific peptide oxidation study on carbon electrodes, showing the oxidation of the amino acids Tyrosine, Tryptophan, Histidine, Cysteine, and Methionine, with the first oxidation of the Tyrosine and Tryptophan residues, followed by oxidation of Histidine, and then half of the benzene tryptophan residues being electrochemically hydroxylated. Only the M3R protein has a satisfactory amount of these amino acids to maximize the anodic peak of DMAA. However, these amino acids have several oxidation windows, and the results obtained at the 0.2 V oxidation potential could only be related to the oxidation of polytyrosine and polytryptophan, eight residues of M3R structure.

Furthermore, using 4-DMAA as an electrochemical indicator, we could only observe its oxidation potential at 0.2 V, corresponding to the nitrogen oxidation of the tertiary amine that is the pyrazole substituent, forming a radical cation [13].

Hu et al. (2010) [23] described two anodic peaks for 4-aminoantipyrine in cyclic voltammetry: 0.56 V and 0.94 V (on carbon glass electrodes, ranging from 0.52 V to 0.91 V on carbon nanotube electrodes), which correspond to the oxidation of the -NH_2_ group and the antipyrine, respectively.

Based on these data and our results, the interaction of 4-DMAA with the peptide probes probably occurs in the region of the aromatic ring of the pyrazole, so we did not identify in the voltammetric tests any anode peak at 0.9V corresponding to the oxidation of the pyrazole. The same is true for the oxidation potential at 0.6 V described for the amine radical, which could also not be seen in these results. We believe that this favorable potential at 0.2 V refers to the oxidation of nitrogen in the dimethylamine substituent group present in 4-DMAA (absent in antipyrine (AA)) and that the absence of the other oxidation potentials found in AA is due to the interactions of the pyrazole group and the aromatic rings with the adsorbed biomolecules and the graphite sensing layer, respectively (Luong et al., 2009), which would also explain the oxidation potentials of 4-DMAA in the 0.7 V range observed in our experiments with a gold electrode.

### 3.4. Topographic Analysis AFM

**Figure 6:**
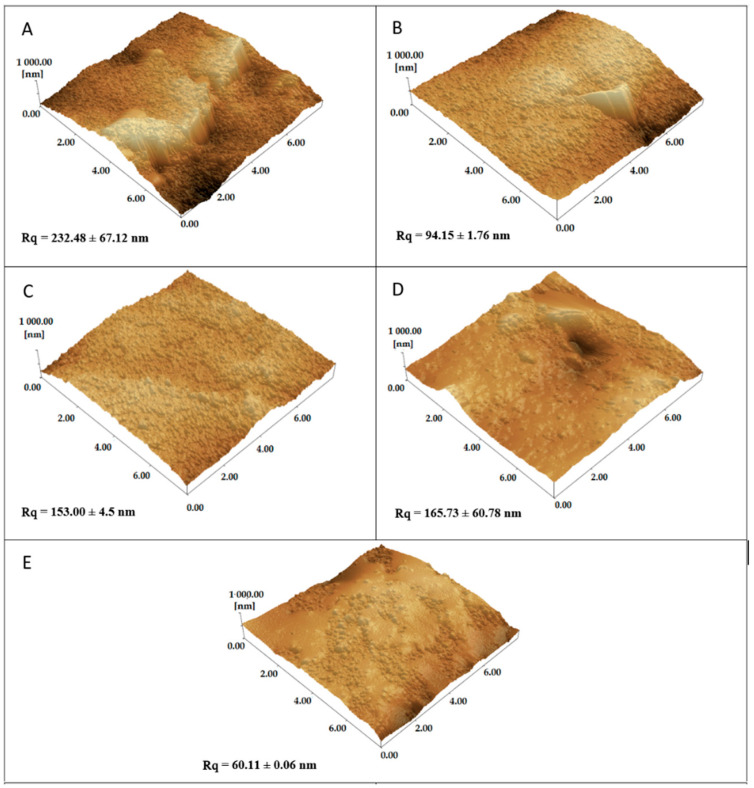
Atomic force microscopy (AFM) images of graphite electrode (C110) (**A**); 4-DMAA added to C110 (**B**); LC1 added to C110 followed by 4-DMAP (**C**); LC1 + 4-DMAP with serum IgG-positive (**D**); LC1 + 4-DMAP with serum IgG-negative (**E**). Rq is equivalent to roughness values.

Surface analyses of the C110 electrode with the best assembly strategy performed for probe 1 (visceral leishmaniasis-specific) are shown in Figure 6. The addition of 4-DMAA on the surface of the graphite (Figure 6B) and the electrode previously functionalized with the peptide probe (Figure 6C) produced a decrease in the height and size of the clusters, characterizing the reduced roughness when compared to the virgin C110 electrode (Figure 6A), suggesting that the indicator and peptide are filling the cavities formed on the graphite surface. Interestingly, the best reduction in graphite surface roughness was observed when 4-DMAA was incorporated alone, suggesting a better planar interaction with the electrode. After adding the positive serum (Figure 6D), the roughness increased significantly (Rq = 165.73) with a globular appearance when compared to the negative serum (Rq = 60.11, Figure 6E), showing the occurrence of agglutination between the probe and the specific IgG.

### 3.5. Additional Considerations

The results showed that 4-dimethylaminoantipyrine is a new and versatile electrochemical indicator for immunosensors based on synthetic peptides and carbon/graphite electrodes, as shown by Al-Memary (2019) and Gangwar (2022). This is explained by its easy adsorption on this surface through the amino groups and the aromatic ring, which also binds to proteins through electrostatic and van der Waals forces. 

4-DMAA oxidizes at low anodic potential and the intensity of the oxidation peak can be useful for detecting immunoglobulins and serum proteins in other biological fluids. This study showed that incorporating 4-DMAA into the electrode before serum IgG allowed the detection of the antigen-antibody recognition of interest on four different probes. The sensor response was mainly dependent on the concentration of the probe when compared to peptides of the same number of amino acids (24 amino acid residues), but the size of the protein molecule used as a probe influenced the intensity of the anode peaks displayed on the DPV.

Although we did not evaluate the influence of pH on the system, we believe that little can be done in this regard, considering the possibility of conformational changes of the probes with pH variation that could be incompatible with the preservation of the recognition sites. We have also used probes previously constructed to recognize specific antibodies in patient sera, but not designed specifically for use in electrodes and electrochemical studies. However, the method used to obtain these probes, phage display, allows us to produce probes with specific sequences to facilitate the adsorption process on the working electrode surface, producing greater homogeneity in results and consequently greater sensitivity in detection.

## 4. Conclusions

4-DMAA can be used as a universal indicator for immunoassays based on amino acid polymers (peptides and proteins). We emphasize that in our study, there was no pretreatment of the working electrode surface and that this tool can also be the subject of future studies for the development of new electrochemical systems and used in future serological detection of other diseases. Therefore, we can conclude that there is a favorable interaction of 4-DMAA with graphite, peptide probes, and with the BSA.

## Figures and Tables

**Table 1:** Target diseases, probes ID and concentrations, serum dilution, and specific references for each probe.

Probe ID	Probe Concentration	Target Disease	Serum Dilution	* IRB Protocol
LC1	38.0 μg.mL^−1^	*Visceral* *leishmaniasis*	1:100	CEP 099/2003, 499/2008
LT	22.5 μg.mL^−1^	*Tegumentary leishmaniasis*	1:100	CEP 099/2003, 499/2008
D3	4.0 μg.mL^−1^	*Strongyloidiasis*	1:160	CEP 553/2009
M3R	3.5 µg.mL^−1^	*Leprosy*	1:100	CEP 449/2010, CAE N.23115003005/2009-36

* Institutional Research Board (UFU), number of the protocol and date.

## Data Availability

Not applicable.
